# Defining usual care: do we need to think beyond clinical equipoise? A synthesis and secondary analysis of multiple qualitative data sets

**DOI:** 10.1186/1745-6215-16-S2-P80

**Published:** 2015-11-16

**Authors:** Katrina Turner, John Percival, Jenny Donovan, David Kessler

**Affiliations:** 1 University of Bristol, Bristol, UK

## 

Pragmatic mental health trials often have a ‘usual care’ arm. To date, no one has explored in detail what this arm actually entails. This presents difficulties for those recruiting individuals to trials and prevents researchers fully appreciating the reasons underlying any differences found between the clinical outcomes of different trial arms.

We synthesized data collected during four qualitative studies, nested within large, primary care depression trials, in order to integrate patients’ experiences of usual care. In all four trials, participants allocated to the intervention arms were provided with a clear description of the prospective treatment, contacted to initiate treatment, and had continuity of care regarding the practitioner they saw. Participants allocated to usual care were simply told to follow their GP’s advice.

We thematically analysed 48 interview transcripts. Participants’ accounts of usual care indicated some individuals felt unable to contact their GP, that GPs varied in the amount of time and support they gave patients, that patients were unclear what treatment options existed, and that many patients experienced a lack of continuity of care. In addition, some individuals felt their GP did not know how to manage their depression.

The findings suggest there are important differences in patients’ experiences of intervention and usual care arms which relate not only to the treatment they receive but also to their experiences of accessing care, how care is delivered, and what treatment and practitioner expectations patients have. These differences need to be taken into account when defining usual care and interpreting trial results.

